# Case Report: Severe psittacosis in an elderly patient without avian exposure: diagnosis via metagenomic next-generation sequencing and rapid response to doxycycline

**DOI:** 10.3389/fmed.2025.1689333

**Published:** 2025-12-18

**Authors:** Xuxin Sun, Qihui Jiao, Xiaoyan Fu, Tingyi Xie, Wei Xie, Hong Cheng, Sheng Chen

**Affiliations:** 1 The Fourth Clinical Medical College of Guangzhou University of Chinese Medicine, Shenzhen, China; 2 Shenzhen Traditional Chinese Medicine Hospital, Shenzhen, China

**Keywords:** *Chlamydia psittaci*, doxycycline, MetaCAP™, metagenomic next-generation sequencing, zoonotic transmission

## Abstract

*Chlamydia psittaci* causes psittacosis in both birds and humans, typically following avian exposure. We present a case of severe psittacosis in a 73-year-old woman with no documented bird contact. The diagnosis was ultimately achieved through metagenomic next-generation sequencing (mNGS) after initial conventional serologic tests failed to identify a pathogen. The patient presented with fever and pneumonia that were unresponsive to broad-spectrum antibiotics. mNGS performed on a whole-blood sample collected on hospital day 5 detected *C. psittaci*, albeit with a low number of specific sequence reads (only 14 reads mapping to the *C. psittaci* genome). Oral doxycycline (100 mg q12h) was initiated promptly, resulting in defervescence within 24 h and resolution of inflammatory markers. Although community pet parrots were identified as a potential source, the patient denied any direct contact. This case highlights the risk of environmental aerosol transmission in the absence of direct avian exposure, demonstrates the critical role of mNGS in diagnosing culture-negative pneumonia, and underscores the efficacy of early doxycycline therapy. Strengthened public health surveillance of avian reservoirs is imperative to mitigate unrecognized transmission.

## Introduction

Psittacosis, a zoonosis caused by *Chlamydia psittaci*, primarily affects avian species. Human infection usually occurs through inhalation of aerosolized secretions from infected birds, with direct contact historically considered necessary for transmission (1). Although it accounts for approximately 1.03% of community-acquired pneumonia cases, its true prevalence is likely underestimated due to diagnostic challenges (2). Typical manifestations include respiratory symptoms such as fever, cough, and dyspnea, which may progress to severe complications including acute respiratory distress syndrome or multi-organ failure (3). These nonspecific features often mimic bacterial pneumonia, leading to diagnostic delays and inappropriate antimicrobial treatment.

This report describes a case of severe psittacosis in a 73-year-old woman with no history of avian exposure. After empirical treatments failed and conventional diagnostics returned negative, the diagnosis was confirmed using metagenomic next-generation sequencing (mNGS). Marked clinical improvement was observed shortly after initiating doxycycline.

### Case presentation

A 73-year-old woman was admitted on June 2, 2025, with a one-day history of fever accompanied by dizziness, generalized malaise, and diaphoresis. Her medical history was unremarkable, with no chronic respiratory, cardiovascular, renal, or metabolic conditions, and she denied recent contact with birds or poultry. Physical examination revealed an alert but fatigued patient with a temperature of 38 °C, blood pressure was 138/86 mmHg, heart rate was 98 bpm, respiratory rate was 18 breaths/min, and oxygen saturation was 96% on room air. Cardiopulmonary and neurological examinations were unremarkable. Empirical intravenous antimicrobial therapy with cefoperazone-sulbactam (1.5 g every 12 h) was commenced.

**Figure 1:**
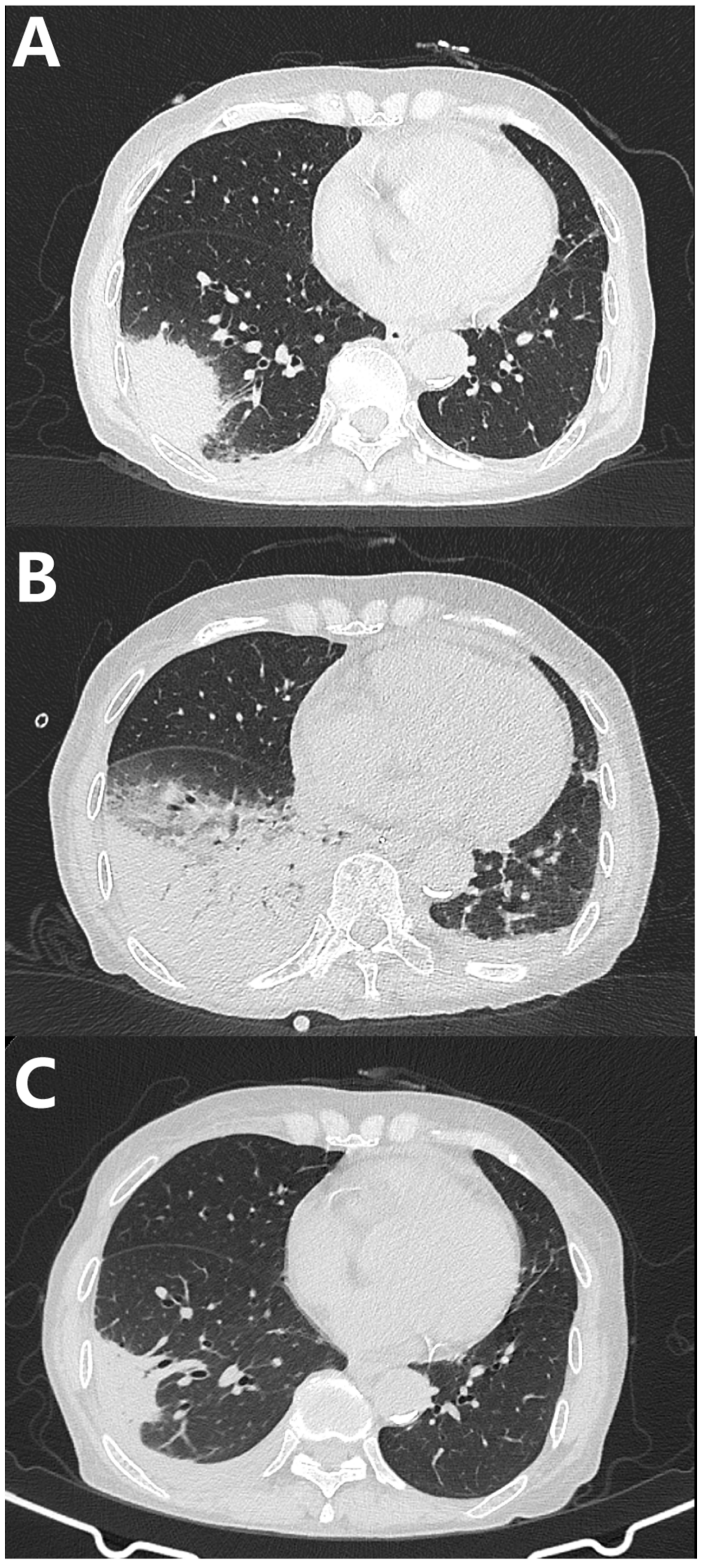
Serial chest CT imaging. **(A)** Initial CT demonstrating right lower lobe consolidation, scattered nodules, and pericardial effusion. **(B)** CT on June 8 showing progression with increased consolidation and new bilateral pleural effusions. **(C)** CT on June 15 demonstrating significant interval resolution.

Initial laboratory tests revealed leukocytosis (WBC 11.92 × 10^9^/L) with marked neutrophilia (87.2%) and an elevated C-reactive protein level (104.69 mg/L). Hyponatremia (136 mmol/L) was observed, although renal and hepatic function were normal. Chest CT showed consolidation in the right lower lobe (etiology indeterminate), scattered pulmonary nodules, a small right pleural effusion, and cardiomegaly with pericardial effusion (Figure 1A).

Despite administration of intramuscular aspirin-DL-lysine (450 mg) and oral ibuprofen (300 mg), her body temperature rose to 39.7 °C on the evening of admission (June 2). Antibiotic therapy was escalated to intravenous moxifloxacin (400 mg once daily) on hospital day 2 (June 3).

On hospital day 3 (June 4), inflammatory markers had increased significantly: CRP rose to 219.77 mg/L and procalcitonin peaked at 9.7 ng/mL. An extensive infectious workup returned negative, including respiratory viral PCR (influenza A/B, respiratory syncytial virus, adenovirus, and parainfluenza viruses), serology for atypical pathogens (*Mycoplasma pneumoniae*, *Legionella pneumophila*, and *Chlamydia pneumoniae*), *Mycobacterium tuberculosis* antibody assays, SARS-CoV-2 nucleic acid amplification testing, and aerobic/anaerobic blood cultures, which showed no growth after 72 h.

Given persistent fever and worsening inflammation despite sequential antibiotic regimens, antimicrobial coverage was broadened on June 4 to dual therapy with moxifloxacin (400 mg q24h) and meropenem (0.5 g q8h). Intravenous immunoglobulin (IVIG; 2.5 g daily for 8 days; total 20 g) was administered as adjunctive immunomodulatory therapy, in consideration of the patient’s progressive clinical deterioration, signs of severe systemic inflammation, and insufficient response to initial broad-spectrum antibacterial agents.

Between June 2 and 6, multiple antipyretics were administered—including intramuscular aspirin-DL-lysine, oral ibuprofen, acetaminophen, and a paracetamol-based combination agent (paracetamol/pseudoephedrine/dexchlorpheniramine)—yet fever control remained inadequate.

Despite days of antimicrobial and immunomodulatory treatment, the patient continued to experience recurrent high fevers. To identify the etiology, metagenomic next-generation sequencing (mNGS; MetaCAP™) was performed on a whole blood sample on hospital day 5 (June 6). Results confirmed *C. psittaci* infection (14 sequence reads) on June 7, establishing a diagnosis of psittacosis. Meropenem was discontinued immediately, oral doxycycline (100 mg q12h) was started, and moxifloxacin was maintained for 48 h as transitional coverage before being discontinued on June 9.

Within 24 h of doxycycline initiation (hospital day 6, June 7), the patient became afebrile. Inflammatory biomarkers improved rapidly: procalcitonin decreased from 9.7 ng/mL (June 4) to 0.849 ng/mL (June 9), CRP declined from 237.76 mg/L (June 4) to 20.1 mg/L (June 15), and D-dimer levels trended downward from 3,540 ng/mL (June 8) to 1,500 ng/mL (June 18).

Serial thoracic imaging tracked disease progression and resolution: CT on June 8 showed progressive right-sided consolidation and new bilateral pleural effusions, while CT on June 15 revealed significant interval resolution of infiltrates and effusions (Figures 1B,C).

Supportive care included albumin infusion (cumulative 60 g) for hypoalbuminemia (nadir 27.2 g/L on June 9) and electrolyte repletion. By June 18 (hospital day 17), inflammatory markers had normalized (CRP 10.7 mg/L; procalcitonin 0.069 ng/mL). The patient was discharged on June 20 with complete resolution of symptoms (see Table 1).

**Table 1:** Clinical laboratory results.

Measure	Reference range	Hospital day 1 (June 2)	Hospital day 2 (June 3)	Hospital day 3 (June 4)	Hospital day 4 (June 5)	Hospital day 5 (June 6)	Hospital day 7 (June 8)	Hospital day 8 (June 9)	Hospital day 11 (June 12)	Hospital day 14 (June 15)	Hospital day 17 (June 18)
WBC count (×10^9^/L)	3.50–9.50	11.92↑	13.21↑	9.50	9.14	8.73	7.84	7.24	8.41	5.70	4.57
Neutrophil count (×10^9^/L)	1.80–6.30	10.40↑	11.9↑	8.82↑	8.30↑	7.71↑	6.03	5.26	6.43↑	3.86	2.41
Neutrophil (%)	40.0–75.0	87.2↑	90.6↑	92.9↑	90.8↑	88.4↑	77.0↑	72.6	76.3↑	67.8	52.7
Hemoglobin (g/L)	115–150	124	113↓	116	104↓	98↓	102↓	104↓	93↓	93↓	90↓
Platelets (×10^9^/L)	125–350	242	229	225	235	255	324	361↑	502↑	555↑	557↑
Myoglobin(ng/ml)	0.0–107.0	132.0↑	141.0↑	/	124.0↑	116.0↑	108.0↑	82.9	60.5	61.8	61.2
D-dimer (ng/ml)	0–600	385	1,370↑	2,930↑	3,000↑	2,990↑	3,540↑	3,510↑	2010↑	1,630↑	1,500↑
Na (mmol/L)	137.0–147.0	136.0↓	133.9↓	133.7↓	132.7↓	135.3↓	129.7↓	130.0↓	126.6↓	134.9↓	136.8↓
K(mmol/L)	3.50–5.30	4.27	4.18	3.03↓	3.21↓	3.27↓	2.88↓	3.63	4.55	4.61	4.68
AST (U/L)	13.0–35.0	/	35.4↑	43.7↑	45.6↑	/	/	94.5↑	49.8↑	34.4	/
Albumin (g/L)	40.0–55.0	/	34.9↓	/		/	/	27.2↓	34.5↓	37.4↓	/
PCT (ng/mL)	<0.046	0.224↑	2.180↑	9.700↑	6.400↑	3.100↑	1.450↑	0.849↑	0.257↑	0.106↑	0.069↑
CRP (mg/L)	0.0–6.0	/	171.0↑	/	/	231.4↑	170.5↑	111.7↑	37.0↑	20.1↑	10.7↑
IL-6 (pg/mL)	0–7.0	/	308.5↑	/	/	111.1↑	59.2↑	44.2↑	26.8↑	12.8↑	10.2↑

WBC, white blood cells; AST, aspartate aminotransferase; PCT, procalcitonin; CRP, C-reactive protein; IL-6, interleukin-6.

↓ The value in the patient was below normal.

↑ The value in the patient was above normal.

**Figure 2:**
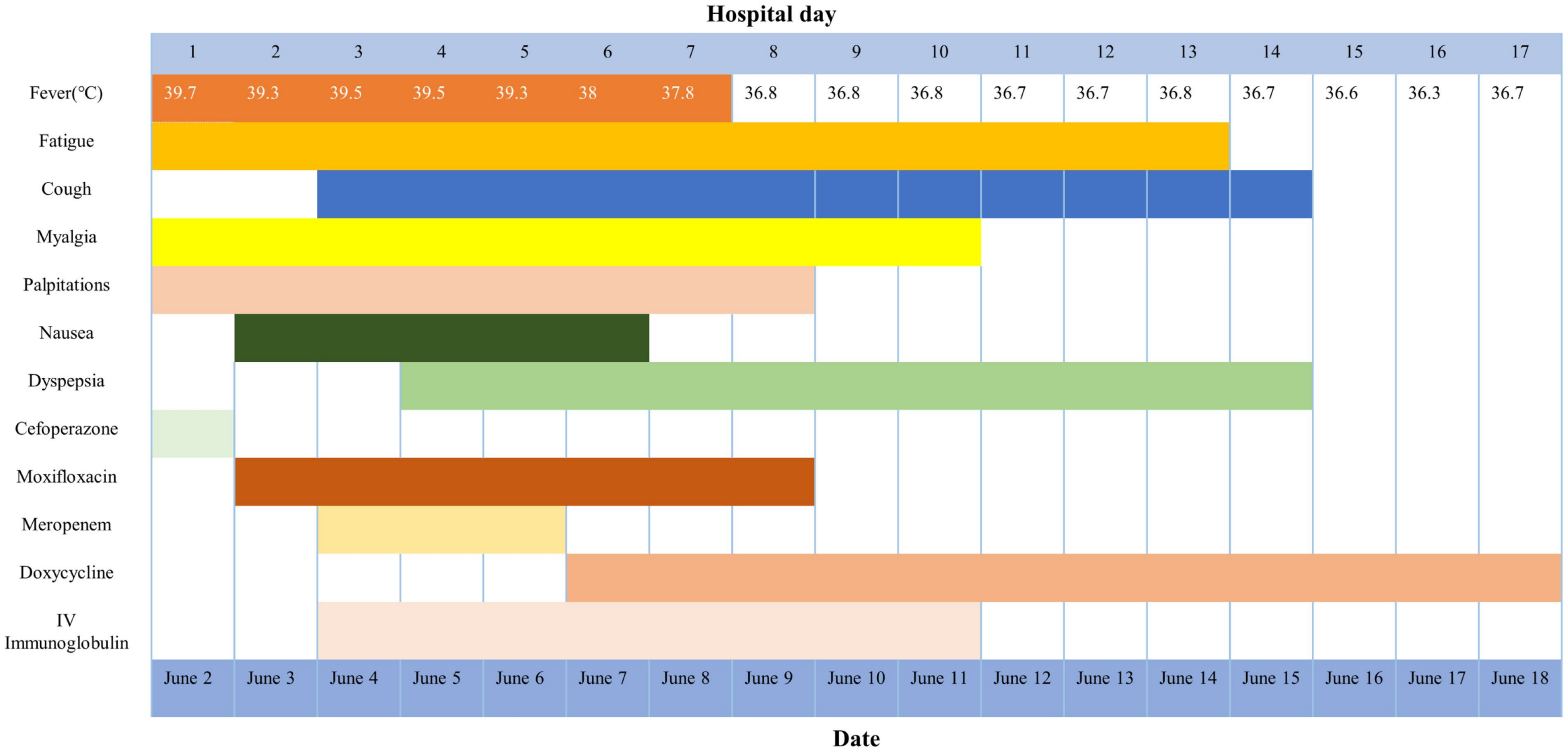
Symptoms, treatment, and maximum body temperatures according to day of illness and day of hospitalization.

A telephone follow-up 2 weeks later (July 4, 2025) confirmed sustained recovery without recurrence. Epidemiological investigation revealed that pet parrots were kept by residents in the same community, although the patient firmly denied any direct contact with these birds or their owners. No additional psittacosis cases were identified in the community (see Figure 2).

## Discussion

This case highlights three key challenges in psittacosis management: the absence of a typical exposure history (2), nonspecific initial manifestations mimicking severe bacterial pneumonia (4), and the limitations of conventional diagnostic techniques (5).

The patient’s unequivocal denial of avian contact underscores a common diagnostic pitfall, even though direct exposure remains the classic epidemiological risk factor. This scenario is not uncommon; a case series highlighted that up to 60% of patients diagnosed with psittacosis report no history of bird contact (6), underscoring the clinical relevance of alternative transmission routes. Recent evidence points to several underrecognized transmission routes that may explain missing exposure histories. Environmental aerosol transmission was plausible given confirmed community-housed parrots near the patient’s residence, indicating that inhalation of contaminated dust or feather particles can occur without direct interaction. Subclinical exposure through transient contacts—such as brief visits to bird markets—may also be sufficient, particularly in elderly patients with potentially compromised recall. This risk is compounded by asymptomatic birds that shed pathogens; studies have shown that 14.3% of pet parrots in Chinese markets tested positive, with budgerigars reaching 21.8% (7). Furthermore, human-to-human transmission via respiratory droplets, once considered rare, has been definitively documented in healthcare and household settings through secondary and tertiary transmission chains (8), challenging the traditional view that psittacosis requires avian exposure. These mechanisms explain why missing exposure history contributed to delayed diagnosis in this case, in which clinicians initially prioritized bacterial pneumonias. Thus, psittacosis should be considered in cases of community-acquired pneumonia even without reported bird contact, particularly in regions with psittacine reservoirs, with mNGS serving as a vital tool for unbiased pathogen detection.

The patient’s clinical picture exemplifies how psittacosis can masquerade as severe bacterial pneumonia. Initial features—including systemic inflammation (fever, leukocytosis 11.92 × 10^9^/L, CRP > 200 mg/L), lobar consolidation on imaging, and rapidly progressive pleural effusions—are hallmarks of bacterial pathogens such as *S. pneumoniae* or *K. pneumoniae*. This mimicry delayed targeted treatment and led to unnecessary antibiotic escalation. Negative serological and targeted PCR results reflect well-known limitations: serology has low sensitivity in early infection, and targeted PCR may fail to detect unexpected pathogens (9). Such diagnostic uncertainty necessitates a systematic search for atypical clues, such as extrapulmonary symptoms, in severe CAP patients not responding to first-line therapy, and warrants expedited molecular testing when conventional tests are negative. This approach proved successful here, as mNGS identified *C. psittaci* within 24 h.

Pathogen-agnostic metagenomic next-generation sequencing (mNGS) of whole blood was performed using the MetaCAP™ platform, a targeted enrichment platform that selectively captures genomic sequences from a comprehensive panel of respiratory and bloodstream pathogens. This technology enhances sensitivity for target pathogens compared to untargeted mNGS, while retaining broad detection capability (10, 11). Despite the low sequence read count (*n* = 14), the specificity of the detection was ensured by the platform’s rigorous bioinformatic pipeline. While independent confirmatory testing (e.g., PCR on a subsequent sample) was not performed following the positive mNGS result, the patient’s rapid and definitive clinical response to doxycycline provides strong corroborative evidence. This case underscores that in complex diagnostic scenarios, mNGS—particularly via optimized platforms like MetaCAP™—can be decisive. Therefore, its application should be considered in cases of severe, antibiotic-refractory community-acquired pneumonia, even in the absence of classic epidemiological clues, to enable timely targeted therapy (12, 13).

The dramatic therapeutic response—defervescence within 24 h of doxycycline initiation and rapid resolution of inflammatory markers—confirms tetracyclines as first-line treatment for psittacosis (14, 15). Although not guided by current guidelines, the 48-h adjunctive use of moxifloxacin may have mitigated risks of bacterial co-infection during diagnostic uncertainty, consistent with bridging strategies in severe pneumonia (16). This case underscores two fundamental principles in psittacosis management: (1) tetracyclines are the therapeutic cornerstone, with rapid defervescence serving as a key diagnostic-therapeutic indicator; and (2) judicious short-course adjunctive therapy (e.g., with respiratory fluoroquinolones) provides a safety net during confirmatory testing, especially in severe cases with potential mixed infections. This case highlights the role of rapid pathogen identification via mNGS in facilitating antimicrobial stewardship, allowing for timely de-escalation from broad empirical regimens to targeted therapy.

This case also highlights a broader public health consideration beyond individual patient diagnosis. While the clinical identification of *C. psittaci* was achieved, the inability to investigate the suspected avian reservoir underscores a common gap in the “One Health” chain of evidence. In ideal practice, a human case of psittacosis without direct exposure should act as a sentinel event, triggering coordinated environmental and veterinary surveillance to trace the source and implement control measures. Strengthening formal channels for collaboration between clinical, public health, and veterinary authorities is therefore crucial. Such integration would not only close the diagnostic loop in individual cases but also enhance community-wide prevention of zoonotic spillover.

## Conclusion

This case underscores that psittacosis is a diagnostic consideration in severe, refractory pneumonia regardless of exposure history. For clinicians, a high index of suspicion coupled with the use of mNGS for rapid pathogen identification is crucial to guide timely, life-saving therapy with doxycycline. Beyond the individual patient, this case highlights the necessity of a proactive “One Health” approach. Effective surveillance and control of this zoonosis require strengthened collaboration between human public health and veterinary sectors to trace avian reservoirs, assess environmental risks, and prevent future outbreaks. Thus, the convergence of advanced diagnostics in human medicine and integrated cross-sectoral surveillance forms the cornerstone of managing emerging zoonotic threats.

## Data Availability

The original contributions presented in the study are included in the article/supplementary material, further inquiries can be directed to the corresponding authors.
